# Inflammatory myofibroblastic tumor of the bladder – an unexpected case coexisting with an ovarian teratoma

**DOI:** 10.1186/1746-1596-9-138

**Published:** 2014-07-15

**Authors:** Zuzanna Dobrosz, Janusz Ryś, Piotr Paleń, Paweł Właszczuk, Marek Ciepiela

**Affiliations:** 1 Department of Histopathology, Medical University of Silesia, Medyków Street 18, Katowice 40-754, Poland; 2 Department of Tumour Pathology, Centre of Oncology – Maria Skłodowska-Curie Memorial Institute, Cracow Branch, Gancarska Street 11, Krakow 31-115, Poland; 3 Department of Obstetrics and Gynaecology, City Hospital No 2, Jastrzębie Zdrój, Poland

**Keywords:** Inflammatory Myofibroblastic Tumor, Bladder neoplasm, Immunohistochemistry, Fish analysis

## Abstract

Inflammatory myofibroblastic tumors (IMTs) mainly occur in children and young adults, usually in the first two decades of life. IMT-type tumors belong to neoplasms of an intermediate biologic potential with considerable rate of local recurrence and in some cases that able to create metastases. Presented case is the first IMT coexisting with the other neoplasm. In our paper we are going to present a peculiar case of an IMT of the bladder coexisting with an ovarian teratoma, and to discuss its pathogenesis, histological picture and differential diagnosis. A 19-year-old female was admitted to the Gynecological Department and during the surgery, two independent, non-adjacent tumors were found. To settle the diagnosis, a FISH examination with the ALK1 break apart probe was carried out. It confirmed the rearrangement of the chromosome 2p23. Morphologic and immunophenotypic similarities between an IMT and other malignant tumors of the bladder may lead to diagnostic errors and an unnecessary radical cystectomy as a result. The therapy of choice is only total excision of the tumor.

**Virtual Slides:**

The virtual slide(s) for this article can be found here: http://www.diagnosticpathology.diagnomx.eu/vs/1937487606122622

## Background

Inflammatory myofibroblastic tumors (IMTs) mainly occur in children and young adults, usually in the first two decades of life. The most frequent sites of these tumors are the lungs, the peritoneum and the mesentery [1,2]. IMTs are very rarely localized in the bladder, so they need to be differentiated from other bladder tumors [3], mainly rhabdomyosarcomas and leiomyosarcomas.

Tumors of non-epithelial origin account for about 2-5% of all neoplasmatic tumors of the bladder: rhabdomyosarcomas appear most frequently in children under the age of ten, while leiomyosarcomas are most frequent in adults [4]. In our paper we are going to present a peculiar case of an IMT coexisting with an ovarian teratoma, and discuss its pathogenesis, histological picture and differential diagnosis.

## Case report

A 19-year-old female was admitted to the Gynecological Department of the Municipal Hospital due to 3-month-long pain in the hypogastrium. An ultrasonographic study revealed a tumor on the left ovary corresponding to a teratoma with hypoechogenic structure; it just seemed that the tumor infiltrated the wall of the bladder and grew into its lumen. However, during the surgery, two independent, non-adjacent tumors were found. One of these was a tumor on the left ovary, 45 mm in diameter. The tumor had a well-defined capsule, and macroscopically was diagnosed as a teratoma. The second lesion was a hard tumor 30 × 40 mm, well circumscribed, which was deforming the anterior wall of the bladder, and growing into its lumen. The left appendices and the uterus were normal. An insignificant amount of transudate fluid was found on the pelvic floor. The affected ovary was excised, and then, the anterior wall of the bladder was incised and a solid tumor of 40 × 30 mm was removed.

**Figure 1:**
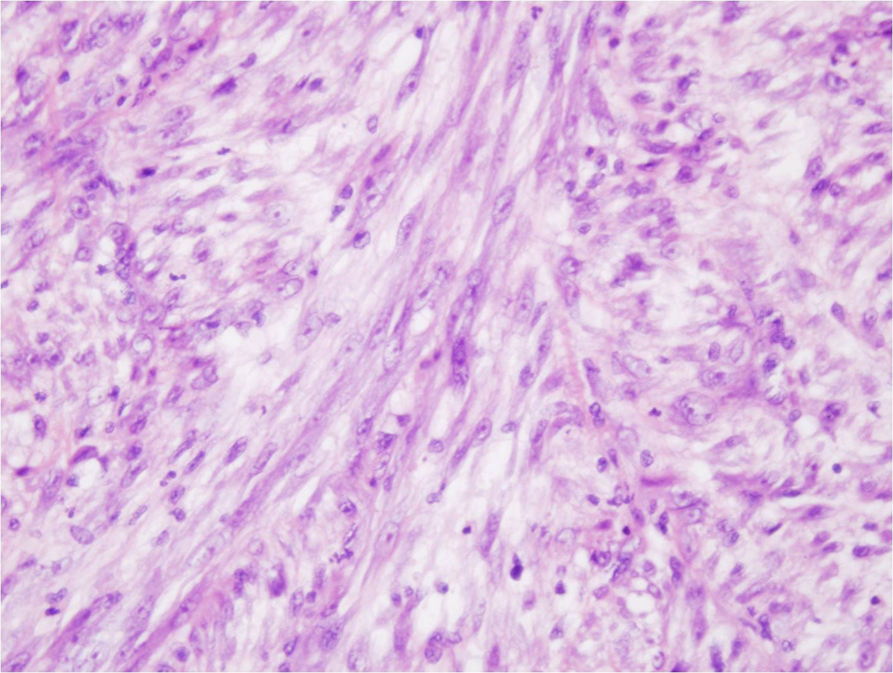
HE staining.

**Figure 2:**
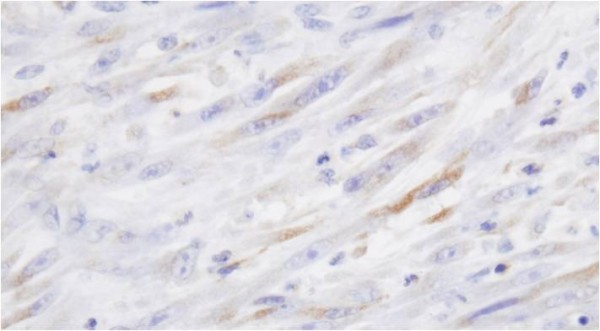
Calponine staining.

**Figure 3:**
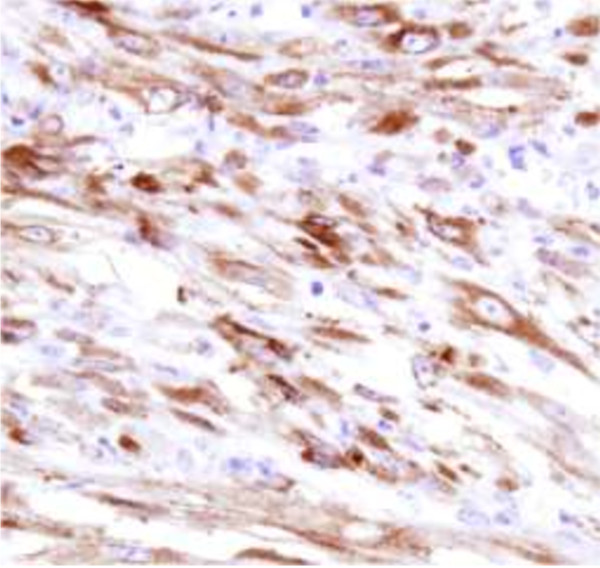
Alk-1 staining.

**Figure 4:**
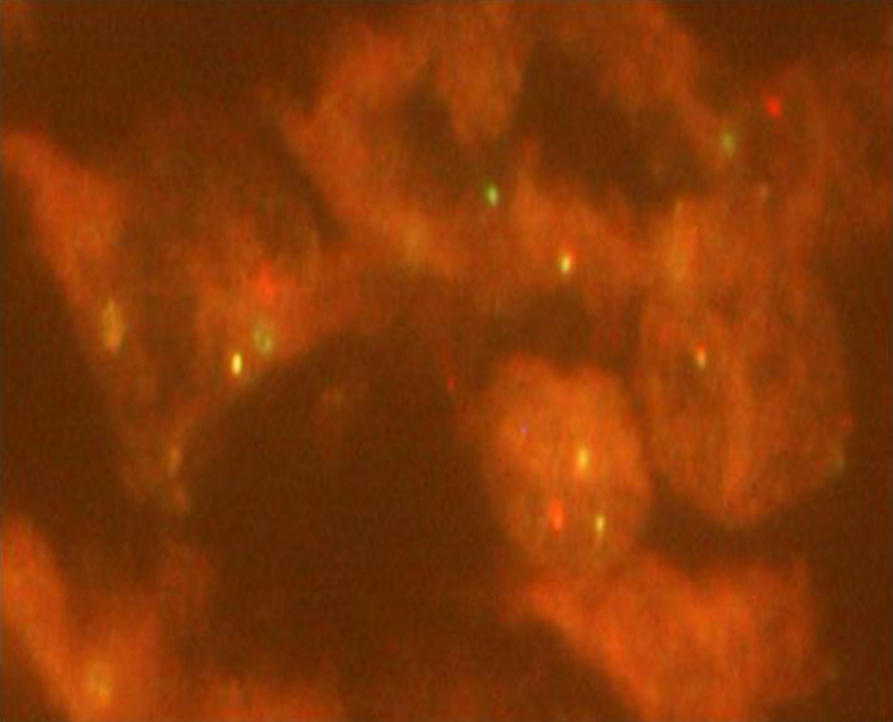
FISH analysis.

Histologically, the bladder tumor was well circumscribed and built of bundles of spindle cells divided by abundant amounts of myxoid extracellular matrix. The tumor cells featured long cytoplasmatic processes and vesicular or elongated nuclei with one or several delicate nucleoli (Figure 1). They present a moderate proliferative activity that means up to three mitoses per one high power field, the Ki-67 index was about 15%, and S-phase fraction about 19.9%. The bladder transitional epithelium, bordering the tumor, was normal. Immunochemically, the tumor’s cells were positive for cytokeratins (AE1/AE3) (Table 1), smooth muscle actin, calponin (Figure 2), smooth muscle actin, Alk-1 antigen (Figure 3), and focally also for desmin. The immunohistochemical reactions against MyoD1, CD30, CD34 and miogenin (Myf-4) were negative. To settle the diagnosis, a FISH examination with the *ALK1* break apart probe was carried out. It confirmed the rearrangement of the chromosome 2p23 (Figure 4). Flow cytometry analysis revealed that 67% of cells were aneuploid (DI index was 1.12). The neoplasmatic cells were accompanied by an abundant infiltrate of lymphocytes, plasmocytes and polynuclear granulocytes.

**Table 1:** IHC staining (type of cytokeratines) IMT presented in article

**Cytokeratin**	**Reaction**
CK 7	Negative (-)
CK5/6	Negative (-)
CK 20	Negative (-)
CK 8	Negative (-)
CK 18	Negative (-)
CK 19	Negative (-)
AE1/AE3	Positive (+)

## Discussion

An IMT is a relatively rare tumor. In the past, it was described as a plasma cell granuloma, an inflammatory proliferation of myofibroblasts or an inflammatory pseudotumor – IPT. An IMT is usually found in children and young adults, slightly more often in female (F:M = 4:3) and localized mainly in the lungs, however it may arise in almost every location. IMTs situated in the bladder are very rare [2,5-12]. To our knowledge, the presented case is the first IMT coexisting with the other neoplasm. The only exception was a case of an IMT in a patient with Recklinghausen disease, another with systemic lupus erythematous, and the next one with Wolf- Hirschhorn syndrome [6,9,13] (Table 2).

**Table 2:** Cases of urinary bladder IMT

**No of case**	**Sex**	**Age**	**Size**	**Clinical symptoms**	**Immunophenotype of tumor cells**	**First diagnosis**	**FISH**	**Follow up**	**No of (art.)**
1	M	13	6 × 3 cm	Weight loss, hematuria, abdominal pain	CK(+), VIM(+), ALK-1(+)	Proliferation of fusiform myoepithelial cells	Nd	2 months without recurrence	5
2	F	27	6.4 × 4.5 × 3,8 cm	Fever, diffuse myalgia, weight loss, anemia,	CK(-), SMA(+), MyoD1(-), ALK-1(+)	IMT	Nd	2 months without recurrence	6
3	F	35	7.5 × 4.5 cm	Duration, gross hematuria, bacteruria	CK(-), VIM(+), S-100(-), CD68(+)	Glandular cystitis	Nd	1 year without recurrence	7
4	F	36	2.4 × 2.3 × 2.2 cm	Hematuria, abdominal pain	CK8/18/19 focally(+), S100(-),ALK1(+), MyoD1(-), CD117(-)	Low grade leiomyosarcoma	Nd	3 years without recurrence	8
5	M	58	3 cm	hematuria,	CK(-), VIM(+)	Postoperative spindle cell nodule (PSCN) but excluded because of absence of previous surgery	Nd	Nd	9
6	F	27	6 × 5 cm	Hematuria, dots in urine, burning micturition, weakness, abdominal pain	CK 20(-), SMA(+), VIM(+), ALK-1(+), DES(-), CD117(-)	IMT	Nd	15 months without recurrence	10
7	M	30		Nd	CK(-),SMA(+), VIM(+), ALK-1(+), DES(+), Myogenin(-)	IMT	Nd	Nd	11
8	F	52	3 cm	Painful urination	CK(+), ALK-1(+)	Sarcomatoid carcinoma	Nd	1 year without recurrence	12
9	F	8	4.5 cm	Hematuria, abdominal pain	SMA(+), VIM(+), ALK-1(+), DES(+), Myoglobin(-), CD34 (-), S-100 (-)	IMT	Nd	13 months without recurrence	13

Nd- no date available.

The first case of an IMT in the bladder was described by Roth in 1980 [14]. The origin of this tumor was a matter of a long debate; some authors maintained that an IMT is a consequence of an inflammation in the bladder, while others regarded it as a neoplastic lesion. The Epsten-Barr virus, the human herpes virus HHV8, bacteria such as *Campylobacter equi*, *Campylobacter jejuni*, *Escherichia coli*, trauma, radio- and steroidotherapy were considered to be causative factors [1,2]. In some articles, Hepatitis C, HIV and TB infections prior to the development of pseudotumors have been documented [2,15]. Fangusaro at al. described two cases of IMT following hematopoetic stem cell transplantation. Both patients received total body irradiation in preparation for the transplant. These two cases suggest radiation as a possible underlying cause of IMT [16]. However, more recent research has showed that an IMT is most probably a neoplasm rather than an inflammatory pseudotumor; both rearrangement of 2p23 chromosome as well as sporadic local invasion or metastases speak in favor of neoplasmatic origin of the tumor [17,18].

Teratomas are neoplasms that arise from pluripotent cells and can differentiate along one or more embryonic germ lines [19,20]. Mature teratomas of the ovary are one of the most common benign ovarian neoplasms, accounting for approximately 10-20% of all ovarian tumors. Teratoma may occur at any age in women, but predominantly occurs in younger patients (20–40 years old) [21]. Mature ovarian teratomas are benign ovarian germ cell tumors that usually occur with a normal karyotype. There are very few reports describing chromosomal abnormalities in these tumors, none of which are recurrent. The Ding Y study in 2011 was the first to demonstrate the differential profile of 16 miRNAs in mature ovarian teratomas. An aberrant expression of miRNAs may be essential for the pathogenesis of mature ovarian teratomas [22].

An IMT may occur in the bladder at any age (from childhood to old age) [23,24]. This rare disease does not usually cause pain; the most frequent symptoms are severe hematuria, sometimes anuria and a palpable tumor. The tumor may be localized in any region of the bladder, however the trigone of the bladder has never been affected, apart of the cases where the tumor arose in the posterior wall of the bladder and secondarily infiltrated the trigone [23]. The size of the tumor is variable – from several centimeters up to 37.5 cm [25]. Obviously, it is not possible to distinguish an IMT from malignant tumors of the bladder with diagnostic methods such as an RTG or endoscopy. Macroscopically, these tumors are well circumscribed, light and soft; the cut surface is smooth, occasionally opalescent, without hemorrhagic foci or necrosis [25].

Histologically, it is possible to distinguish three types of these tumors: the vascular-myxoidal type, which can resemble nodular fasciitis or granulomatous tissue; the solid, spindle-cell type, which resembles a fibrous histiocytoma or tumor originating from smooth muscle or a low-cellular-fibrous, which imitates desmoids or a scar [2,8,26].

The key criteria in IMT diagnostics are: proliferation of myoepithelial spindle cells accompanied by lymphocytic infiltration of tumor stroma, the positive immunohistochemical reaction to ALK-1, vimentin and cytokeratin [27,28], and finally *ALK1* gene rearrangement confirmed cytogenetically or by FISH method. It shows the rearrangement of the ALK gene in the population of spindle cells in an IMT [26]. The ALK gene is located on the 2p23 chromosome; it encodes the ALK protein, the tyrosine kinase receptor. This method is a very sensitive tool used to differentiate an IMT from other spindle-cell tumors of the bladder. In IMT tumors, the positive immunohistochemical reaction with an antibody against ALK-1 is observed in more than 60% cases, while in the FISH method it is observed in almost 70% of the cases. Overexpression of this gene is also observed in anaplastic large cell lymphoma [29]. Some IMTs show an expression of cytokeratins, SMA or desmin [18,27]. Myogenin, a rhabdomyosarcoma marker, allows this tumor to be excluded [30].

Morphologic and immunophenotypic similarities between an IMT and other malignant tumors of the bladder may lead to diagnostic errors and an unnecessary radical cystectomy as a result.

The misdiagnosis of an IMT as a rhabdomyosarcoma, a leiomyosarcoma or a sarcomatoid urothelial carcinoma, and as a result, unnecessary radical surgery, adjuvant therapy and its complications, is a major problem of contemporary IMT diagnostics.

## Conclusions

IMT-type tumors belong to neoplasms of an intermediate biologic potential with a considerable rate of local recurrence and in some cases they are able to create metastases [31]. In the case described in this paper, the presence of a large necrosis in the central portion of the tumor, mitotic activity and the presence of aneuploidal cells (seen in cytofluorometry) suggest a more aggressive type of IMT. The therapy of choice is the total excision of the tumor, a radical cystectomy is not necessary [32,33].

### Consent

The study (No 1340143-46) was performed in accordance with the Declaration of Helsinki and the protocol was approved by the local Human Research Ethics Committee. Informed consent was obtained from the patient for publication of this case report and any accompanying images.

## Competing interest

The authors declare that they have no competing interests.

## Authors’ contribution

ZD: main author, author diagnosis and preparing of the manuscript. JR: carried out the molecular genetic studies. PW: participated in the sequence alignment and drafted the manuscript. PP: collecting literature. MC: medical history and clinical course of the case.
